# High-resolution patterning of colloidal quantum dots via non-destructive, light-driven ligand crosslinking

**DOI:** 10.1038/s41467-020-16652-4

**Published:** 2020-06-08

**Authors:** Jeehye Yang, Donghyo Hahm, Kyunghwan Kim, Seunghyun Rhee, Myeongjae Lee, Seunghan Kim, Jun Hyuk Chang, Hye Won Park, Jaehoon Lim, Minkyoung Lee, Hyeokjun Kim, Joohee Bang, Hyungju Ahn, Jeong Ho Cho, Jeonghun Kwak, BongSoo Kim, Changhee Lee, Wan Ki Bae, Moon Sung Kang

**Affiliations:** 1 Department of Chemical and Biomolecular Engineering, Sogang University, Seoul, 04107 Republic of Korea; 2 SKKU Advanced Institute of Nanotechnology (SAINT), School of Nano Science & Technology, Sungkyunkwan University (SKKU), Suwon, 16419 Republic of Korea; 3 Department of Electrical and Computer Engineering, Inter-University Semiconductor Research Center, Seoul National University, Seoul, 08826 Republic of Korea; 4 Department of Chemistry, Korea University, Seoul, 02841 Republic of Korea; 5 Department of Energy Science, Center for Artificial Atoms, Sungkyunkwan University (SKKU), Suwon, 16419 Republic of Korea; 6 Pohang Accelerator Laboratory, POSTECH, Pohang, 37673 Republic of Korea; 7 Department of Chemical and Biomolecular Engineering, Yonsei University, Seoul, 03722 Republic of Korea; 8 Department of Chemistry, Ulsan National Institute of Science and Technology (UNIST), Ulsan, 44919 Republic of Korea

**Keywords:** Quantum dots, Electronic properties and materials, Quantum dots

## Abstract

Establishing multi-colour patterning technology for colloidal quantum dots is critical for realising high-resolution displays based on the material. Here, we report a solution-based processing method to form patterns of quantum dots using a light-driven ligand crosslinker, ethane-1,2-diyl bis(4-azido-2,3,5,6-tetrafluorobenzoate). The crosslinker with two azide end groups can interlock the ligands of neighbouring quantum dots upon exposure to UV, yielding chemically robust quantum dot films. Exploiting the light-driven crosslinking process, different colour CdSe-based core-shell quantum dots can be photo-patterned; quantum dot patterns of red, green and blue primary colours with a sub-pixel size of 4 μm × 16 μm, corresponding to a resolution of >1400 pixels per inch, are demonstrated. The process is non-destructive, such that photoluminescence and electroluminescence characteristics of quantum dot films are preserved after crosslinking. We demonstrate that red crosslinked quantum dot light-emitting diodes exhibiting an external quantum efficiency as high as 14.6% can be obtained.

## Abstract (web summary)

Designing high-resolution displays based on colloidal quantum dots remains a challenge. Here, the authors demonstrate a photo-patterning method to develop CdSe-based core-shell quantum dots patterns of red, green and blue colours with diameters ranging from 7 to 20 nm and resolution of 1400 pixels per inch.

## Introduction

Colloidal quantum dots (QDs), nanocrystalline semiconductors with dimensions residing in the quantum confinement regime^1–4^, exhibit ultrahigh colour purity and near-unity luminescence quantum yield^5–7^. These excellent characteristics of QDs have led the materials to be successfully incorporated into commercial display devices, positioned to challenge organic light-emitting diode (OLED) technology. These commercial devices, as of now, harness the photoluminescence (PL) characteristics of QDs; PL from QDs in these devices contributes to forming a high-quality white light source for the display, whereby the white light is split into primary colours through liquid crystal layers and colour filters before being conceived by the human eye^8–10^. The technology is now driven to launch displays based on the electroluminescence (EL) of QDs^11–22^, akin to what has been achieved from OLEDs. Among the issues to be resolved, the development of processing methods to precisely locate red (*R*), green (*G*) and blue (*B*) QDs at a given position in the pixel over a large area is one of the critical challenges in realising EL displays based on QDs. The challenge arises from the fact that QDs are processed from solutions, unlike organic luminophores used for OLEDs that are typically processed by thermal evaporation^23–25^. While the solution processability of QDs allows low-cost production of films over a large area, it prevents conducting a secondary solution process on top of the underlying QD film. This indicates that conventional photoresist-based patterning methods are hardly applicable, unless the surface of QDs is delicately managed^26^; the QD films would be soluble to the solvent used to apply the photoresist. Furthermore, forming patterns of different colour QDs (e.g., *R*, *G*, *B* patterns of QDs) side-by-side by conducting consecutive cycles of solution processing is challenging, because processing one of the QD layers is likely to damage the underlying QD patterns. Han and colleagues^27^ employed an additional layer of positively charged polyelectrolyte underneath the film of QDs modified with negatively charged ligands. High-resolution patterns of QDs could be successfully prepared, but the luminescence characteristics of QDs could not be preserved completely. Alternative patterning methods for QD films have been developed extensively, including ink-jet printing^28–30^ and micro-contact printing^31–33^. These methods, however still require further development for industrial-scale usage in terms of the achievable uniformity, resolution and throughput rate.

The utilisation of light-driven chemical/physical transformation of QD films for patterning is a promising strategy that can meet these practical requirements. Manna and colleagues^34^ demonstrated that aliphatic ligands of QDs can be activated under X-ray exposure to form chemically crosslinked QD films. A similar approach was done by Liao and colleagues^35^ using an Ar plasma as the irradiation source. Despite the success of patterning, the use of a high-energy X-ray or plasma source is likely to cause loss of PL, which prevents the use of this process for luminescent applications. Talapin and colleagues^36,37^ designed inorganic ligand molecules anchored on the surface of QDs, which can be transformed upon exposure to various wavelengths of ultraviolet (UV)–Visible (Vis) light (254–450 nm) and even to *e*-beam. As the solubility of the QD film alters as the surface properties of ligand molecules change under irradiation, QD film could be patterned by selectively removing either the irradiated region or the un-exposed region with an appropriate developer solvent. Consequently, they demonstrated micrometre-sized QD patterns and multi-layered patterns of *RGB* QDs by repeating the patterning process. However, the luminescence properties of the resulting QD patterns have not been investigated comprehensively, which are critical to their optical or optoelectronic applications.

Here, we report a simple yet effective method to form high-resolution patterns of QDs that preserves the inherent luminescent properties of the material using a light-driven ligand crosslinker (LiXer). UV exposure on a blended film prepared from QD-LiXer mixed solutions galvanises the chemical reaction between azides and the alkyl chain of QD surface ligands to construct a chemically robust QD network. Because of the excellent crosslinking efficiency of fluorinated phenyl azides we used^38–40^, QD patterns are readily achieved with a small amount of LiXer (less than 5 wt%) using a handheld UV-lamp (254 nm, 0.4 mW cm^−2^) over a short period of time (5 s). As the resulting crosslinked QD films are structurally robust against subsequent solution processes, multiple patterns of QDs can be formed through consecutive cycles of solution-based film deposition and photo-patterning processes. Based on this strategy, we successfully fabricate QD line patterns with a minimum feature size of 3 μm and *RGB* QD patterns with a sub-pixel size of 4 μm × 16 μm that corresponds to a resolution of >1400 pixels per inch (p.p.i.). Owing to the little contents of LiXer and benign processing conditions, degradation in the PL characteristics of QDs during the patterning process and the associated EL characteristics of the QD-LEDs could be avoided. Consequently, QD-LEDs yielding an external quantum efficiency (E.Q.E.) of 14.6% could be obtained from the crosslinked QD layer, which is a comparable value achievable from pristine QD layer. The simple strategy presented here will make a significant impact on the production of high-resolution, large area, full-colour QD-LEDs, which are intensively explored across the scientific community to industry.

## Results

### Description of the photo-patterning method based on LiXer

**Fig. 1:**
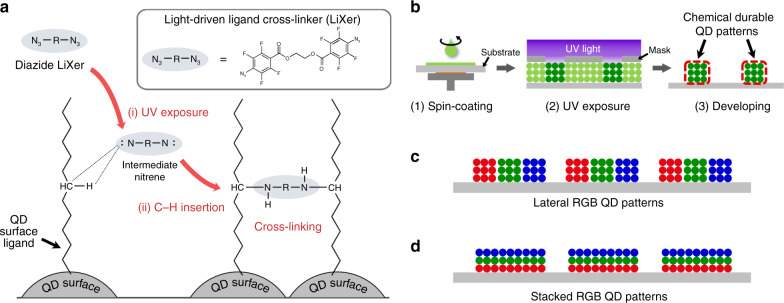
Schematic illustration of QD patterns using LiXer. **a** Schematic description of the ligand crosslinking process between neighbouring QDs based on the C–H insertion reaction of the nitrene moiety of LiXer. The inset shows the chemical structure of ethane-1,2-diyl bis(4-azido-2,3,5,6-tetrafluorobenzoate), used as the LiXer in this study. **b** Schematic description of the photo-patterning processes of QDs using LiXer. **c** Laterally positioned patterns and **d** vertically stacked patterns of *RGB* QDs, achievable from consecutive applications of the photo-patterning process.

Figure 1 describes the core of the high-resolution photo-patterning method for QDs. The method utilises ethane-1,2-diyl bis(4-azido-2,3,5,6-tetrafluorobenzoate) as the LiXer that contains two fluorinated perfluorophenyl azide groups at both ends of the molecule^41–44^. The chemical structure of ethane-1,2-diyl bis(4-azido-2,3,5,6-tetrafluorobenzoate) is shown in Fig. 1a. Fluorinated aryl azide is a well-known photo-active moiety forming reactive nitrene intermediate upon exposure to UV (254 nm), which can easily undergo C–H insertion reaction in the presence of alkyl chains nearby^45–47^. In our scheme, the crosslinker with two fluorinated phenyl azide terminals is intended to undergo C–H insertion reaction into the long aliphatic chains of the ligands (i.e., oleic acids or alkyl thiols) that passivate the surface of QDs. Therefore, it allows crosslinking the ligands of neighbouring QDs under exposure to UV. Unlike previous methods^36,37^, the new method can directly utilise high-quality QDs typically terminated with long alkyl chains without undergoing additional ligand modification, which often degrades the luminescence quantum yield of the materials. The patterns of QDs can be formed by (i) simple spin-coating of a solution mixture of QDs and LiXer onto a substrate, (ii) irradiation of UV and (iii) simple developing step using conventional organic solvents (Fig. 1b), all of which are well-established procedures used for photolithography in the semiconductor industry. Moreover, owing to the crosslinked nature of the resulting QD films, they should be chemically robust against subsequent solution processing, even using the very same solvent that was used to cast the given QD films. This allows for a large degree of freedom in realising QD patterns of multiple colours. Since the processing of different coloured QDs can be conducted repeatedly as many times as needed, lateral patterns of *RGB* QDs (Fig. 1c) as well as the vertical or tandem stacking of different QD layers (Fig. 1d) should also be possible by repeating the procedures.

**Fig. 2:**
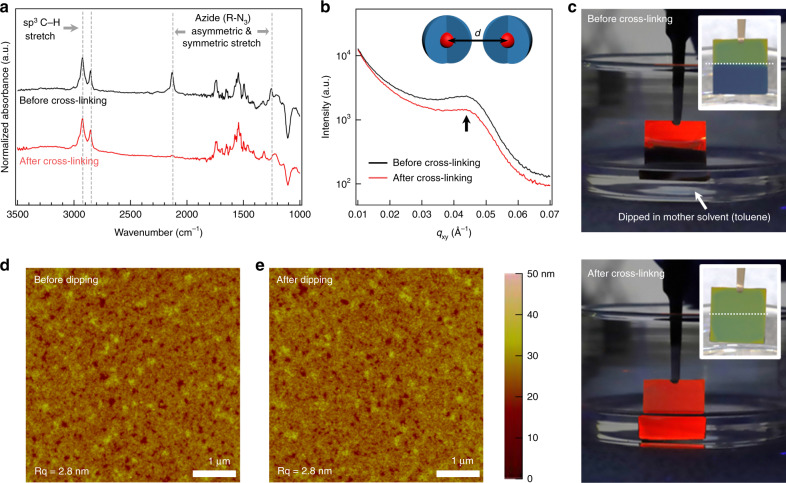
Characterisation of crosslinked QD films prepared with LiXer. **a** FT-IR spectra and **b** GI-SAXS patterns of 13-nm sized CdSe/CdZnS QD films blended with LiXer (5 wt%) before (black) versus after (red) UV exposure (254 nm, 0.4 mW cm^−2^). The FT-IR spectra were normalised to the maximum absorbance of the sp^3^ C–H stretch peak at 2924 cm^−1^ and displayed with an offset for easier comparison. **c** Photographs of a crosslinked QD film with presence of 5 wt% LiXer (bottom) and a pristine QD film (top). Both films were partially dipped into toluene, which is the mother solvent that was used to cast these QD films. AFM images of a crosslinked 13-nm sized CdSe/CdZnS QD film **d** before and **e** after being dipped into toluene.

Figure 2a shows a series of Fourier-transform infrared spectroscopy (FT-IR) spectra for QD films containing the LiXer that was prepared by spin-casting a mixture of 13-nm sized CdSe/CdZnS QDs and LiXer (5 wt%) in toluene. Transmission electron microscope (TEM) images, X-ray diffractograms and absorbance/PL spectra of the QDs used in this work are shown in Supplementary Figs. 1, 2 and 3, respectively. The black spectrum is from the film obtained before it was exposed to UV. The peaks at 2130 cm^−1^ and 1250 cm^−1^ reflect the presence of the N_3_-moieties in the mixture film. The red spectrum is obtained from the same film after UV exposure to a handheld UV source (254 nm, 0.4 mW cm^−2^). Clearly, the peaks associated with the N_3_-moieties were reduced after the exposure to UV, suggesting that chemical reaction described in Fig. 1a has proceeded. Although the formation of the reactive nitrene intermediate and the final secondary amine moiety resulting from the C–H insertion reaction could not be confirmed directly from the FT-IR spectrum^43^, we conjecture that the N_3_-moieties of LiXer underwent the C–H insertion reaction with the long alkyl ligands of neighbouring QDs, which formed the chemically crosslinked QD assembly. Quantitative description on the photon-to-crosslink conversion process of LiXer is provided in Supplementary Information (Supplementary Fig. 4 and Supplementary Discussion). The crosslinking did not reduce the interparticle distance between the QDs. As shown in Fig. 2b, the GI-SAXS pattern of a 13-nm sized CdSe/CdZnS QD added with LiXer (5 wt%) but not exposed to a UV source showed a peak at *q*_xy_ = 0.04386 Å^−1^, corresponding to the mean centre-to-centre distance (*d*) for neighbouring QDs of 14.3 nm. The same film that underwent the crosslinking reaction (red) also showed a peak with a similar *q*_xy_ value (0.04394 Å^−1^), indicating that no discernible change in *d* has occurred upon the photo-crosslinking process.

Crosslinking the ligands awards the ability to withstand a structural failure against consecutive solution processing to QD films. Figure 2c shows the photographs of the crosslinked versus pristine QD films when dipped into the toluene. Note that toluene was the mother solvent that was used to cast the QD film. The submerged part of the pristine QD film dissolved immediately upon dipping to toluene. By contrast, noticeable change in the crosslinked film was not found even after dipping for a long period (Fig. 2d, e). This supports the idea to devise multi-coloured QD patterns either placed side-by-side or stacked vertically simply by repeating the photo-crosslinking reaction of QD films with LiXer and rinsing steps. In fact, only 2 wt% of LiXer in 13-nm sized CdSe/CdZnS QD solution was sufficient to ensure the structural robustness of the QD film.

### Lateral and tandem pattering of QDs

**Fig. 3:**
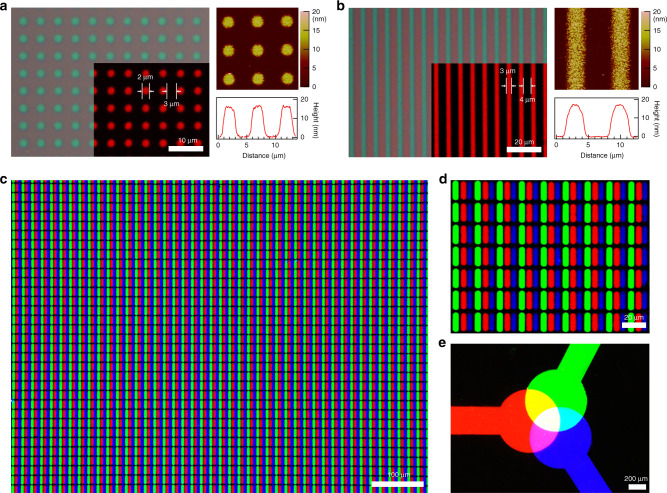
Optical and fluorescence images of QD patterns. Optical and fluorescence (inset) microscopic images and AFM images with height profile of **a** dot (diameter = 2 μm, spacing 3 μm) and **b** line patterns (width = 3 μm, spacing 4 μm) based on 13-nm sized red-emitting CdSe/CdZnS QDs. **c**–**e** Fluorescence microscopic images of *RGB* QD patterns that were obtained through consecutive photo-patterning processes using *RGB* QDs. The dimension of a sub-pixel in Fig. 3c, d is 4 μm × 16 μm, corresponding to a resolution >1400 p.p.i. Fig. 3d is a magnified view of Fig. 3c.

By exploiting the structural robustness of the crosslinked QD films, we could fabricate QD patterns by selective UV exposure through a patterned photomask. Figure 3a, b show sets of optical and fluorescence (inset) images of dot and line patterns based on red QDs, respectively. The patterns were achieved by (i) spin-coating the mixture solution of QD and LiXer in toluene onto a Si/SiO_2_ wafer, (ii) exposing the film to UV irradiation (254 nm, 0.4 mW cm^−2^) through a photomask and (iii) removing the uncrosslinked regime of the film by rinsing with toluene. Dot patterns with a diameter of 2 μm and a spacing of 3 μm as well as line patterns with a width of 3 μm and a spacing of 4 μm could be successfully attained (atomic force microscopy (AFM) image and height profile in Fig. 3a, b). The line edge roughness (LER) of these line patterns was 0.14 μm (Supplementary Fig. 5a). Dot and line patterns could also be formed based on green-emitting CdSe/CdZnSeS QDs (Supplementary Fig. 6). LER of these green-emitting QD line patterns was 0.15 μm (Supplementary Fig. 5b).

Repeated cycles of QD film deposition, photo-patterning and rinsing processes allowed to achieve well-defined high-resolution patterns of different colour QDs. In fact, one should remember that the development of scalable processing methods to precisely locate *RGB* QDs in designated positions within a given pixel is one of the critical challenges in realising EL displays based on QDs. Figure 3c, d displays fluorescence images of the resulting *RGB* QD patterns after repeating photo-patterning steps. The size of a single pattern in the image is 4 µm by 16 µm. The three *RGB* patterns with a spacing of 2 µm constitute a single 18 µm by 18 µm *RGB* square pixel, which corresponds to >1400 p.p.i. in terms of the display pixel resolution. This value is higher than the value that is currently used in commercial OLED displays. In addition to the patterns placed side-by-side, exploiting the structural robustness of the crosslinked QD film allowed the vertical stacking of different colour patterns of QDs. Figure 3e shows a fluorescence image of “the three primary colours of light” expressed through *RGB* QD patterns, formed by repeating the photo-crosslinking process three times. The resulting pattern nicely demonstrates that the photo-crosslinked QD films can be stacked vertically, as well as horizontally. Furthermore, μm-thick layers of QDs can be produced by repeating multiple rounds of film-casting and photo-crosslinking steps, which can be exploited for colour conversion layer applications of QDs (Supplementary Fig. 7).

### Photophysical properties of crosslinked QDs with LiXer

**Fig. 4:**
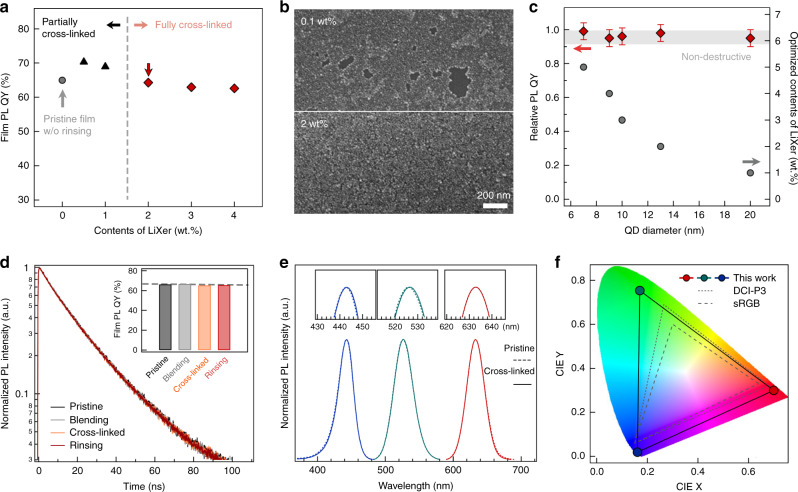
Photophysical properties of crosslinked QD films. **a** Film PL QYs of 13-nm sized red-emitting CdSe/CdZnS QD films as a function of added LiXer contents. **b** SEM images of crosslinked 13-nm sized red CdSe/CdZnS QD films with 0.1 wt% (top) and 2 wt% (bottom) LiXer after rinsing. **c** The optimum LiXer contents for different sized red-emitting QDs (grey circle) and relative PL QYs of crosslinked QD films prepared with the optimum LiXer contents (red square) in comparison with pristine QD films. Error bar represents the standard deviation of the data collected from >5 samples prepared independently. **d** PL decay curves of 13-nm sized red-emitting CdSe/CdZnS QD films with 2 wt% of LiXer throughout the film spin-casting (grey), crosslinking (orange) and rinsing (red) steps. PL decay curve of the pristine QD film (black) is overlaid for comparison. Inset: film PL QYs of each films. Each decay curve was normalised to its maximum count value. **e** PL spectra of pristine (dashed line) and crosslinked (solid line) red-, green- and blue-QD films. The PL QY for the pristine red-, green- and blue-QD films is 65%, 64% and 80%, respectively. Each spectrum was normalised to its maximum PL intensity. Insets expand each spectrum at their peaks. **f** The colour coordinates of red-, green- and blue- QD films in the 1931 CIE chromaticity diagram along with standard colour spaces (sRGB, DCI-P3).

The adoption of reactive radicals in patterning QD films is often regarded as a double-edged sword. The low activation energy to generate radicals with high reactivity enables effective chemical crosslinking of QD solids at a mild reaction condition, but simultaneously the reactive radicals could attack the QD surface and create surface trap states, leading to the reduction of PL QY of the crosslinked films^48^. Given that the reaction of azide radicals with the surface of QDs is primarily responsible for the drawback, optimising the content of LiXer in QD films is acute in achieving well-defined QD patterns with high luminance efficiency. Figure 4a shows PL QYs of 13-nm sized red**-**emitting CdSe/CdZnS QD films as a function of added LiXer contents. QD films prepared with LiXer contents below 2 wt% are readily devastated during the rinsing processes (Fig. 4b). LiXer contents >2 wt% are sufficient to form a robust network of QDs, but high LiXer contents accompany PL QY loss of QD films during the photo-crosslinking (Fig. 4a). To avoid unwanted outcome by the presence of excess azide radicals, we cut the content of LiXer in QD solutions down to the limit that ensures the structural robustness of QD films against rinsing steps. The optimum LiXer content varies depending on the QD dimension; greater LiXer contents for smaller QDs and smaller LiXer contents for larger QDs (Fig. 4c and corresponding PL decay curves in Supplementary Fig. 8). QD films with the optimum content of LiXer retain their PL spectra (peak emission wavelength and the spectral linewidth) and PL QY throughout the photo-crosslinking and the rinsing steps (Fig. 4d, e), representing that the present approach is indeed non-destructive; the photophysical properties of the QD materials could be well-preserved. Resulting QD films emit colour-saturated primary colours solely originating from the band-edge transition in QDs. We note that the colour space achieved with the resulting *RGB* spectra of crosslinked QD films far surpasses the standards of the up-to-date commercial displays (sRGB or DCI-P3) (Fig. 4f).

### Electroluminescence characteristics of crosslinked QDs with LiXer

**Fig. 5:**
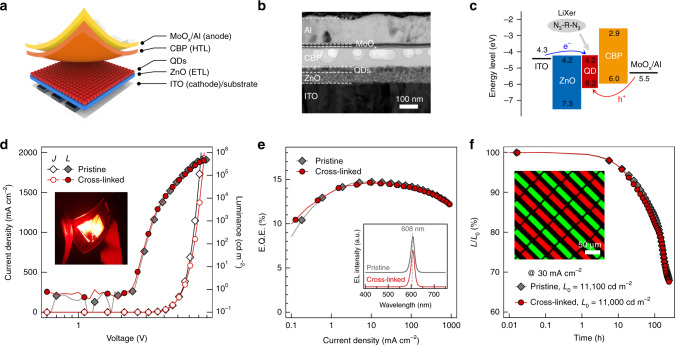
Device characteristics of QD-LEDs with the crosslinked QD emissive layer. **a** Schematic illustration, **b** cross-sectional TEM image and **c** the energy band diagram of a representative QD-LED employing the crosslinked QD emissive layer. **d** Current density (*J)*–voltage (*V)*–luminance (*L)*, **e** external quantum efficiency (E.Q.E.)-*J* and **f** temporal change in relative luminance of QD-LEDs employing pristine (grey diamond) and crosslinked (red circle). *L*_0_ is the luminance at initial. The inset in Fig. 5d is a photograph of a working device prepared on a flexible substrate. The inset in Fig. 5e shows EL spectra of each device. The EL spectrum of a pristine QD-LED is vertically shifted for visual clarity. The inset in Fig. 5f is an EL image of a pixelated *RG* QD-LED (the electrodes as well as the charge injection layers were used in common to operate the device with photo-patterned QD layer). The size of a single rectangular pattern in the image is 10 μm × 38 μm.

As an ultimate achievement, we exemplify QD-LEDs with the photo-crosslinked QD films. The devices were constructed in an inverted structure employing hybrid charge transport layers (Fig. 5a)^16^. For effective electron injection from the indium tin oxide (ITO) cathode to the QD layer, a transparent zinc oxide nanoparticle (ZnO NP) layer was adopted as an electron transport layer (ETL). The 4,4-bis(N-carbazolyl)-1,1-biphenyl (CBP) layer was used as the hole transport layer (HTL) for holes injected from the MoO_3_/Al anode. A 30 nm-thick crosslinked QD emissive layer was prepared from red**-**emitting CdSe/CdZnSe/ZnSeS QD (diameter = 20 nm) dispersion containing 1 wt% of LiXer. Figure 5b shows a cross-sectional TEM image of the QD-LED and Fig. 5c depicts the energy diagram of the device at a static condition (*V*_applied_ = 0 V). We note that the deposition of the QD emission layer, UV crosslinking and rinsing, were all carried out in an inert atmosphere. Figure 5d–f compare the performances of QD-LEDs incorporating a pristine QD layer (black) versus a crosslinked QD layer (red). Apparently, no significant difference was observed in the electrical and optoelectronic characteristics of devices. Specifically, the turn-on voltage (*V*_T_, defined as the voltage yielding at 1 nit) and the peak E.Q.E. of both red-emitting devices were identical as 2.4 V and 14.6%, respectively. (Fig. 5d, e). The peak position of EL spectra (*λ*_EL, max_) and full-width-at-half-maximum (FWHM) of the EL spectra for both the pristine and crosslinked QD-LEDs also matched well. The performance of green- and blue-emitting QD-LEDs, as well as their EL spectra could also be preserved upon exploiting the crosslinked emissive layer (Supplementary Fig. 9). In addition, both red-emitting devices displayed similar device operation stability under the same applied current density (30 mA cm^−2^) (Fig. 5f). These results coherently attest that the emission and electrical characteristics of the QD films are well-preserved even after they are photo-crosslinked, and hence imply that the present approach is indeed applicable to nearly all optical and optoelectronic applications requiring patterned QD arrays. Finally, *RG* pixelated QD-LED were successfully fabricated through consecutive the photo-crosslinking process. (inset in Fig. 5f) The dimension of a single rectangular pattern in the image is 10 μm × 38 μm. This result demonstrates the possibility of realising QD EL displays using LiXer.

## Discussion

A straightforward and effective photo-patterning method of colloidal QDs is reported. QD films with LiXer, which is designed to crosslink the alkyl ligands of neighbouring QDs in films upon exposure to UV, are awarded to retain their structure against exposure to subsequent solution processes. Well-defined QD patterns with feature sizes of a few micrometres are, thus, easily attained by conducting the process cycle of QD/LiXer film deposition, UV exposure and rinsing. Ultimately, tandem processing of the present method permits us to achieve laterally resolved *RGB* QD patterns (>1400 p.p.i.), as well as vertically stacked *RGB* layers at a high resolution. Unlike previous photo-patterning methods, suppression in the PL characteristics of QDs during the patterning process and the associated EL characteristics of the QD-LEDs can be avoided. As only mixing between QDs and LiXer is required, the method should be valid to nearly all QDs that entail alkyl ligands without any delicate secondary steps to prepare a precursor solution for patterning. Thus, the highest quality QDs, holding high luminescence efficiency prepared from state-of-the-art synthetic methods, should be easily applicable to this method. Overall, the approach here is expected to catalyse the practicable use of QDs in production of high-resolution, large-area down-conversion or EL displays.

## Methods

### Materials for synthesis

Zinc acetate (Zn(ac)_2_, 99.99%), sulphur (S, 99.99%), selenium (Se, 99.99%), oleic acid (OA, 99%) and 1-octadecene (ODE, 99%) were purchased from Uniam. *n*-Trioctylphosphine (TOP, technical grade, 90%), 1-dodecanethiol (DDT, 98%), ethylene glycol and dichloromethane (anhydrous, >99.8%) were purchased from Sigma Aldrich. Myristic acid (MA, 90%), cadmium oxide (CdO, 99.9%) were purchased from Alfa Aesar. 4-Azido-2,3,5,6-tetrafluorobenzoic acid (>98%) and triethyl amine (TEA, 99%) were purchased from TCI. All chemicals are used without further purification. Thionyl chloride and the rest of other solvents were purchased from Daejung and used as received.

### Materials for device fabrication

4,4-Bis(N-carbazolyl)-1,1-biphenyl (CBP, 99.9%) were purchased from OSM. Molybdenum oxide (MoO_3_, 99.95%) and aluminium (Al, 99.999%) metal source were purchased from Taewon Scientific Co. (TASCO).

### Synthesis of LiXer

The synthesis of LiXer (ethane-1,2-diyl bis(4-azido-2,3,5,6-tetrafluorobenzoate)) was done by modifying the process described previously by Keana and colleagues^41^. 4-Azido-2,3,5,6-tetrafluorobenzoic acid (931.6 mg, 3.9626 mmol) and thionyl chloride (1 M in dichloromethane) (23.8 mL, 23.77 mmol) were dissolved in anhydrous dichloromethane (30.6 mL), and this solution was heated for 19 h at 70 °C. The reaction mixture was cooled down to room temperature, and then the organic solvents were removed by distillation at a reduced pressure. The resulting acyl chloride compound was dissolved in anhydrous dichloromethane (12 mL) and transferred dropwise to a mixture of ethylene glycol (102.48 mg, 1.6511 mmol) and TEA (400.975 mg, 0.8507 mmol) in anhydrous dichloromethane (18 mL). This reaction mixture was stirred for 12 h at room temperature and quenched by the addition of 1-M HCl_(aq)_ (25 mL). The resulting solution was extracted with dichloromethane (16 mL × 3). The dichloromethane phases were washed with brine (60 mL) and dried over anhydrous MgSO_4_. After filtration, the organic solvent was removed from the filtrate using a rotary evaporator at a reduced pressure. The obtained crude product was purified by silica gel column chromatography, using an ethyl acetate/*n*-hexane (1/5 to 1/3) eluent. Pure white solid of LiXer was obtained (439 mg, 54%). ^1^H-NMR (400 MHz, CDCl_3_): *δ* = 4.68 (s, 4H); ^19^F-NMR (376 MHz, CDCl_3_): *δ* = −138.17 to −138.26 (m), −150.71 to −150.81 (m); ^13^C-NMR (100 MHz, CDCl_3_): *δ* = 159.11‒159.01, 146.90‒146.67, 144.33‒144.10, 141.82‒141.58, 139.32‒139.09, 123.96‒123-72, 107.09‒106.79, 63.39; gas chromatography (GC)/mass spectroscopy (MS) calculated for C_16_H_4_F_8_N_6_O_4_ M^+^: 496.0166, found: *m*/*z* 496.1. Nuclear magnetic resonance (NMR) and GC/MS spectra of LiXer are given in Supplementary Information. (Supplementary Figs. 10–14)

### Synthesis of red-emitting QDs

Stock solutions of 0.5-M cadmium oleate (Cd(OA)_2_) diluted in ODE, 0.5-M zinc oleate (Zn(OA)_2_) in ODE, 2-M TOPSe and 2-M TOPS were prepared for use in the synthesis of QDs. CdSe/CdZnS QDs were synthesised referring to the method used by Lim et al*.*^49^ with minor modification. For a typical synthesis of CdSe/CdZnS QDs, a mixture of 1 mmol of CdO, 3 mmol of MA and 15 mL of ODE were degassed at 110 °C for 2 h, followed by heating to 270 °C to form a clear solution. Meanwhile, 0.25 mL of 2-M TOPSe solution was rapidly injected into the reaction flask and reacted for 3 min at 300 °C to the formation of CdSe cores. To grow the CdZnS shell, 4 mL of 0.5-M Zn(OA)_2_ solution was added to the flask before 1.5 mmol of DDT was added dropwise. After 30 min of reaction at the elevated temperature, Cd(OA)_2_, Zn(OA)_2_ and TOPS (total 8 mmol, 17 mmol and 23 mmol, respectively) were injected repeatedly to grow CdZnS shell in a desired thickness. Synthesised QDs was purified repeatedly via typical precipitation/redispersion method over ten times with various anti-solvents (e.g., ethanol and acetone) before use. Weakly bound TOP on the surface QDs is likely to be removed during this step. 20-nm sized CdSe/CdZnSe/ZnSeS QDs with composition gradient in the two shell layers were synthesised referring to the method used by Lim et al.^18^ CdZnSe shell was grown on CdSe cores by continuous injection of Cd(OA)_2_, Zn(OA)_2_ and TOPSe. CdSe cores were prepared via the method analogous to the recipe for the core of CdSe/CdZnS QDs. For growth of CdZnSe shell, 56 mL of 0.5-M Zn(OA)_2_ solution was added to the flask followed by the continuous injection of a mixture of 12 mL of 0.5-M Cd(OA)_2_ solution, 12 mL of 2-M TOPSe solution and 24 mL of ODE with injection rate of 24 mL h^−1^. Additional ZnSeS shell was grown on the exterior of CdSe/CdZnSe QDs by continuous injection of 80 mmol of Zn(OA)_2_, 32 mmol of TOPSe and 34 mmol of TOPS. Synthesised CdSe/CdZnSe/ZnSeS QDs were purified repeatedly via typical precipitation/redispersion method. Synthetic schemes for green and blue coloured QDs are detailed in Supplementary Methods.

### Device fabrication

Photo-patterned QD-LEDs were fabricated in an inverted structure. Typical processes are described as follows. Glass substrates pre-patterned with indium tin oxide (ITO) electrodes were first cleaned with isopropyl alcohol, acetone and distilled water in an ultrasonic bath for 15 min each. Then, 20 mg mL^−1^ of ZnO NPs dispersed in 1-butanol was spin-coated onto the substrate at 2000 rpm for 40 sec, and the films were annealed at 100 °C for 30 min under a nitrogen atmosphere. Dispersions of QDs in toluene added with different contents of LiXer were spin-coated at 4000 rpm for 30 sec. The resulting films were irradiated with a handheld UV-light source (254 nm, 0.4 mW cm^−2^) for 5 sec to derive the crosslinking reaction between QDs and LiXer. After the irradiation process, the films were developed by rinsing the films with toluene. This process effectively removed the QDs in the areas not exposed to UV (Supplementary Fig. 15). CBP (60 nm), MoO_3_ (10 nm) and Al (130 nm) were consecutively deposited using a thermal evaporator.

### Characterisation

^1^H-NMR, ^13^C-NMR and ^19^F-NMR were measured by a Bruker Avance III HD at 400, 100 and 376 MHz, respectively, with deuterated chloroform (CDCl_3_) as solvent, which was purchased from Cambridge Isotope Laboratories. GC/MS was measured by a Bruker 450-GC and 320-MS at the UNIST Central Research Facilities Centre (UCRF), Ulsan, Korea. UV–Vis absorption and fluorescence spectra of QD films were measured using a V-770 UV–Vis–NIR spectrophotometer (Jasco) and FS-2 fluorescence spectrometer (Scinco), respectively. The measurements of time-resolved photoluminescence (TRPL) were conducted with time-correlated single photon counting (TCSPC) system from Horiba-Jovin Yvon with resolution of about 100 ps. The samples were excited at 3.06 eV at a repetition rate of 500 kHz. FT-IR spectra were collected in a reflectance mode under ambient condition using an iS10 FT-IR spectrometer (Thermo Fisher Scientific). Samples for FT-IR analysis were prepared on bare Si wafer substrates. AFM images were taken with a Park NX10 AFM system (Park Systems) under ambient conditions through a non-contact mode. The thickness of the crosslinked QD films were measured using a Dektak XT-E stylus profiler. X-ray diffraction (XRD) measurements were performed using an Ultima IV X-ray diffractometer (Rigaku) (*λ*CuK*α*1 = 1.5406 Å, 40 kV, 30 mA). Transmission electron microscopy (TEM) images were collected using a JEM-3010 (JEOL) equipped with a Gatan digital camera (MSC-794) (point resolution of 0.17 nm) at the National Centre for Inter-University Research Facilities (NCIRF), Seoul National University. Scanning electron microscopy (SEM) images were obtained using a GeminiSEM 300 (Zeiss). Electrical characteristics of the photo-crosslinked QD films were analysed using a Keithley 236 source measure unit and a Keithley 2000 multimeter connected to calibrated Si photodiodes. Electroluminescence (EL) spectra were measured with a Konica-Minolta CS-2000 spectroradiometer. High-resolution single and multi-colour patterns of QDs were fabricated by using a Karl Süss MA-6 Mask Aligner. Grazing incidence small-angle X-ray scattering (GI-SAXS) measurements were performed at the 9 A U-SAXS beamline of Pohang Light Source-II (PLS-II) in Republic of Korea. The X-ray coming from the in-vacuum undulator (IVU) are monochromated using Si (111) double crystals and focused at the detector position using K-B type mirror. Two-dimensional (2D) scattering patterns were recorded with a 2D CCD (Rayonix MX170-HS). The wavelength of X-ray and sample-to-detector distance were set to be 1.12 Å and 2.5 m, respectively.

## Supplementary information

Supplementary Information

## Data Availability

All data generated or analysed during this study are included in this article (and its Supplementary Information files).
