# Supplementary material for: High-resolution patterning of colloidal quantum dots via non-destructive, light-driven ligand crosslinking
Source: Nat Commun. 2020 Jun 8;11:2874. doi: 10.1038/s41467-020-16652-4 (PMC7280294; doi:10.1038/s41467-020-16652-4)
Supplement: Supplementary file 1 — Supplementary Information [file 41467_2020_16652_MOESM1_ESM.pdf]

High-resolution Patterning of Colloidal Quantum Dots via Non-destructive, Light-driven Ligand Crosslinking

Yang et al

Supplementary Information

High-resolution Patterning of Colloidal Quantum Dots via Non-destructive, Light-driven Ligand Crosslinking

Jeehye Yang,[1, 9] Donghyo Hahm,[2, 9] Kyunghwan Kim,[3, 9] Seunghyun Rhee,[3] Myeongjae Lee,[4] Seunghan Kim,[1] Jun Hyuk Chang,[2] Hye Won Park,[1†] Jaehoon Lim,[5] Minkyoung Lee,[1] Hyeokjun Kim,[1] Joohee Bang,[6] Hyungju Ahn,[6] Jeong Ho Cho,[7] Jeonghun Kwak,[3] BongSoo Kim,[8] Changhee Lee,[3] Wan Ki Bae,[2,*] and Moon Sung Kang [1,*]

[1] Department of Chemical and Biomolecular Engineering, Sogang University, Seoul 04107, Republic of Korea

[2] SKKU Advanced Institute of Nanotechnology (SAINT), School of Nano Science & Technology, Sungkyunkwan University (SKKU), Suwon 16419, Republic of Korea

[3] Department of Electrical and Computer Engineering, Inter-University Semiconductor Research Center, Seoul National University, Seoul 08826, Republic of Korea

[4] Department of Chemistry, Korea University, Seoul 02841, Republic of Korea

[5] Department of Energy Science, Center for Artificial Atoms, Sungkyunkwan University (SKKU), Suwon 16419, Republic of Korea

[6] Pohang Accelerator Laboratory, POSTECH, Pohang 37673, Republic of Korea

[7] Department of Chemical and Biomolecular Engineering, Yonsei University, Seoul 03722, Republic of Korea

[8] Department of Chemistry, Ulsan National Institute of Science and Technology (UNIST), Ulsan 44919, Republic of Korea

[9] These authors contributed equally to this work

*kangms@sogang.ac.kr, wkbae@skku.edu

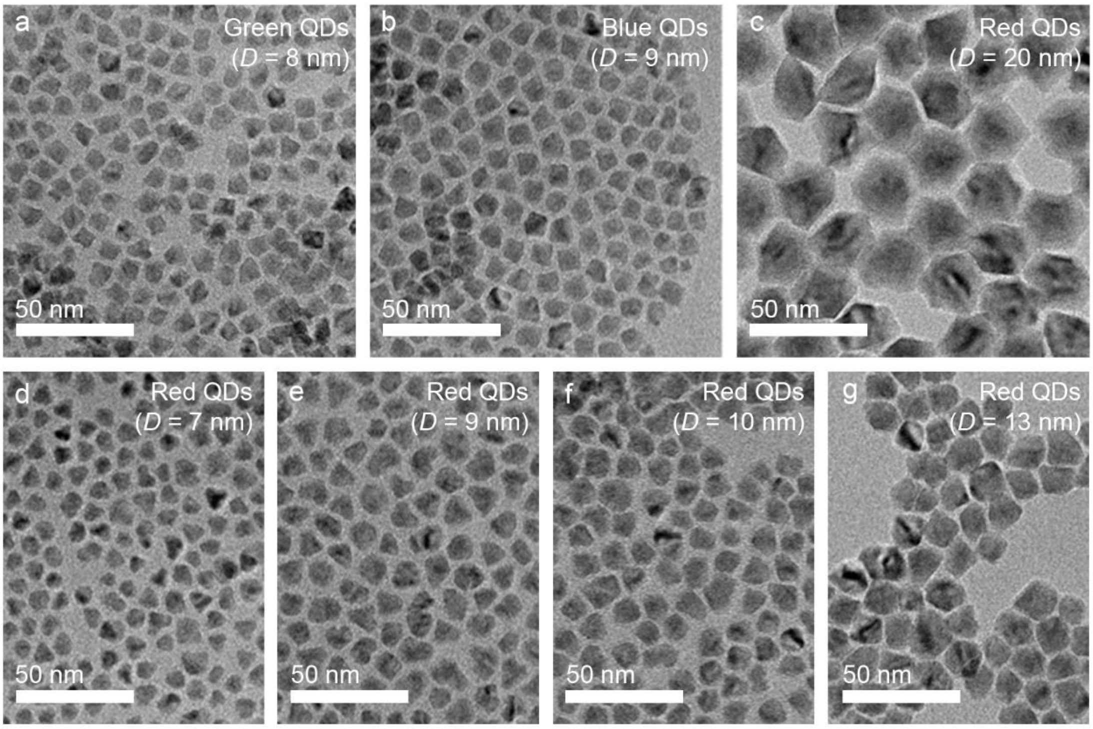

Supplementary Fig. 1 TEM images of *RGB* CdSe-based core-shell QDs used for the experiment a Green-emitting CdSe/CdZnSeS QDs (diameter, $D = 8$ nm, size distribution, $\sigma < 18\%$), **b** blue-emitting CdZnS/ZnS QDs ($D = 9$ nm, $\sigma < 11\%$), **c** red-emitting CdSe/CdZnSe/ZnSeS QDs ($D = 20$ nm, $\sigma < 12\%$), and **d-g** different sized red-emitting CdSe/CdZnS QDs ($D = 7$-13nm, $\sigma < 14\%$).

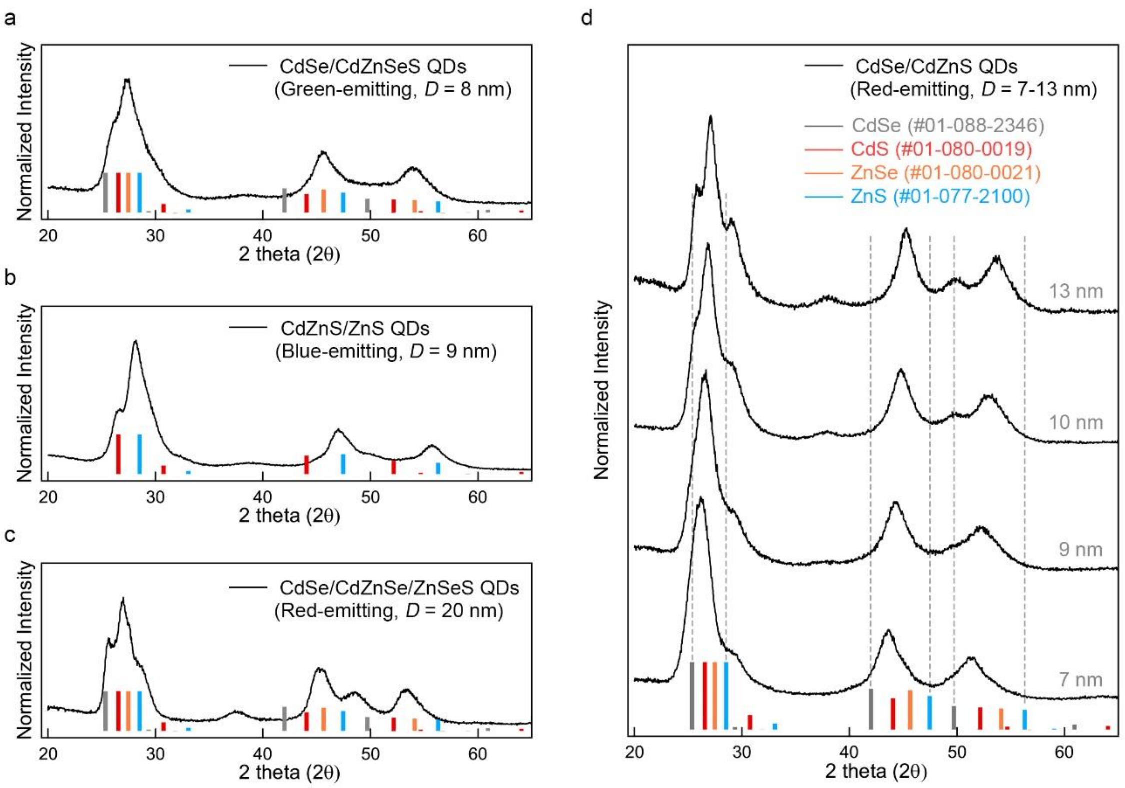

Supplementary Fig. 2 XRD patterns of *RGB* CdSe-based core-shell QDs used for the experiment a 8-nm sized green-emitting CdSe/CdZnSeS QDs, **b** 9-nm sized blue-emitting CdZnS/ZnS QDs, and **c,d** different sized red-emitting CdSe/CdZnSe/ZnSeS QDs (D = 7-20 nm). Standard XRD peaks for CdSe (PDF#01-088-2346, grey line), CdS (PDF#01-080-0019, red line), ZnSe (PDF#01-080-0021, orange line) and ZnS (PDF#01-077-2100, blue line) are indicated at the bottom of each XRD patterns.

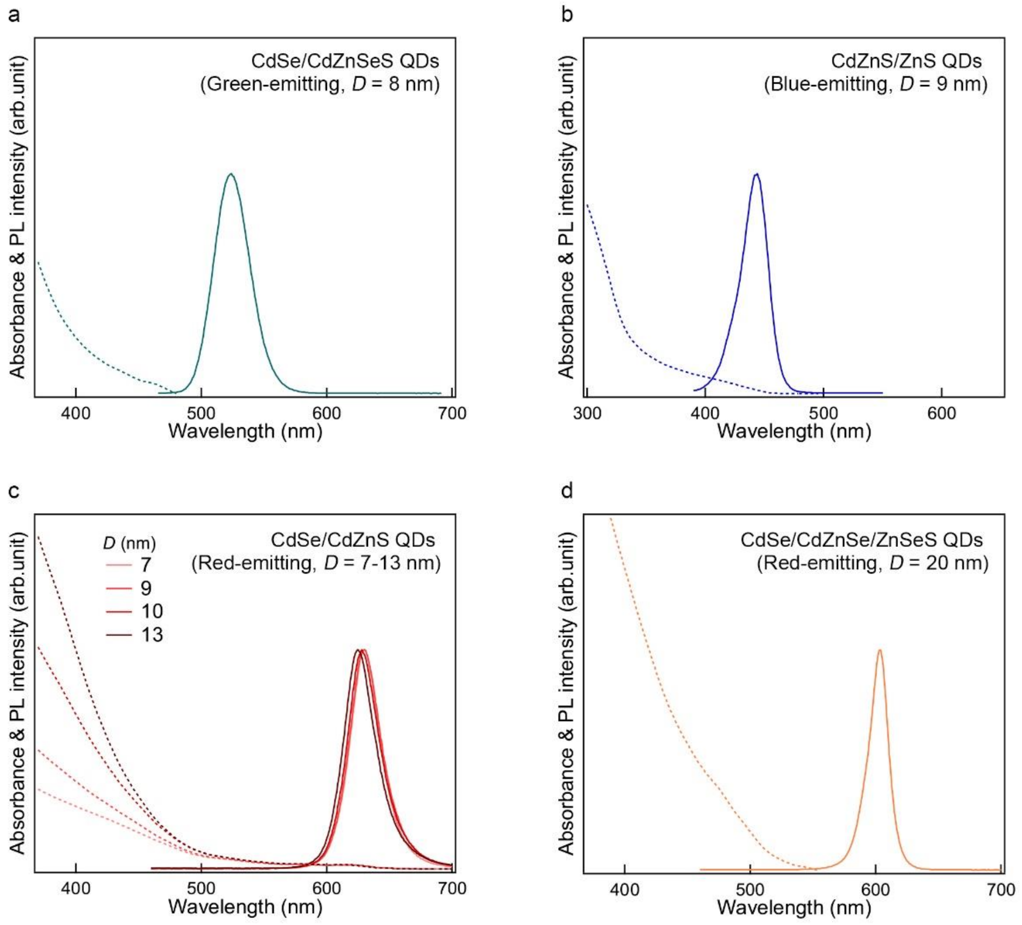

Supplementary Fig. 3 Absorbance and photoluminescence (PL) spectra of *RGB* CdSe-based core-shell QD solutions used for the experiment a 8-nm green-emitting CdSe/CdZnSeS QDs, **b** 9-nm blue-emitting CdZnS/ZnS QDs, **c** different sized red-emitting CdSe/CdZnS QDs (*D* = 7-13nm) and **d** 20-nm red-emitting CdSe/CdZnSe/ZnSeS QDs.

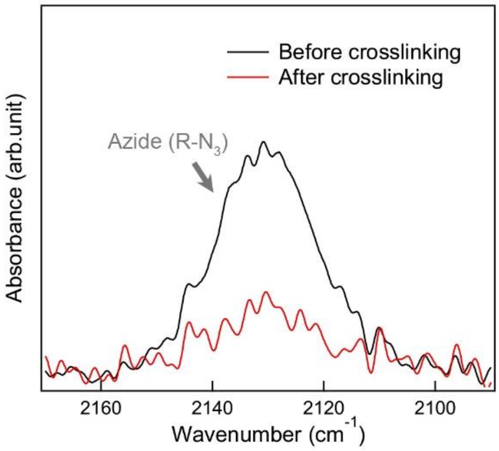

Supplementary Fig. 4 Photo-activation efficiency analysis of fluorinated phenyl azide FT-IR spectra of 13-nm sized CdSe/CdZnS QD films blended with LiXer (2 wt%) before (black) *versus* after (red) UV exposure (254 nm, 0.4 mW cm^{-2}, 5sec, corresponding exposure dose 2mJ cm^{-2}).

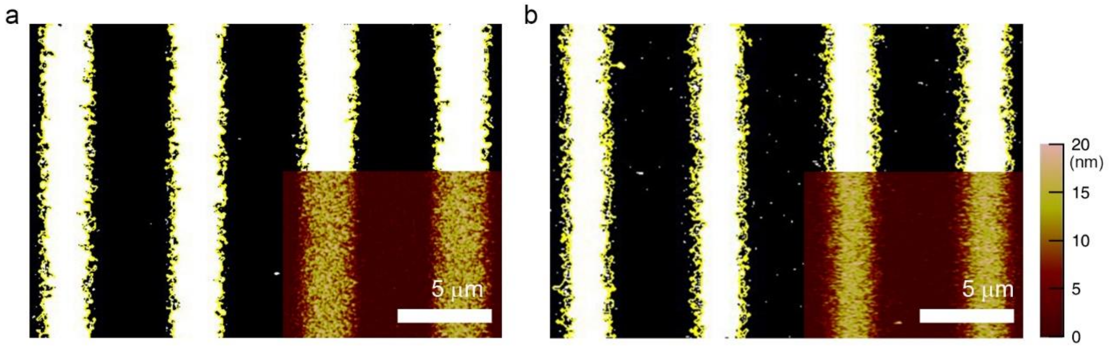

Supplementary Fig. 5 Line edge roughness (LER) analysis of line patterns (3 μm pitch, 4 μm line spacing) a 13-nm sized red-emitting CdSe/CdZnS QDs and **b** 8-nm sized green-emitting CdSe/CdZnSeS QDs were used for *RG* line patterns, respectively. The AFM images are converted to grayscale images to enhance the contrast at the edges of line patterns, which are depicted in yellow. LER of the patterns was analyzed using an image analysis tool, ImageJ. The resulting LERs for line patterns comprising red and green-emitting QDs were measured to be 0.14 and 0.15 μm, respectively.

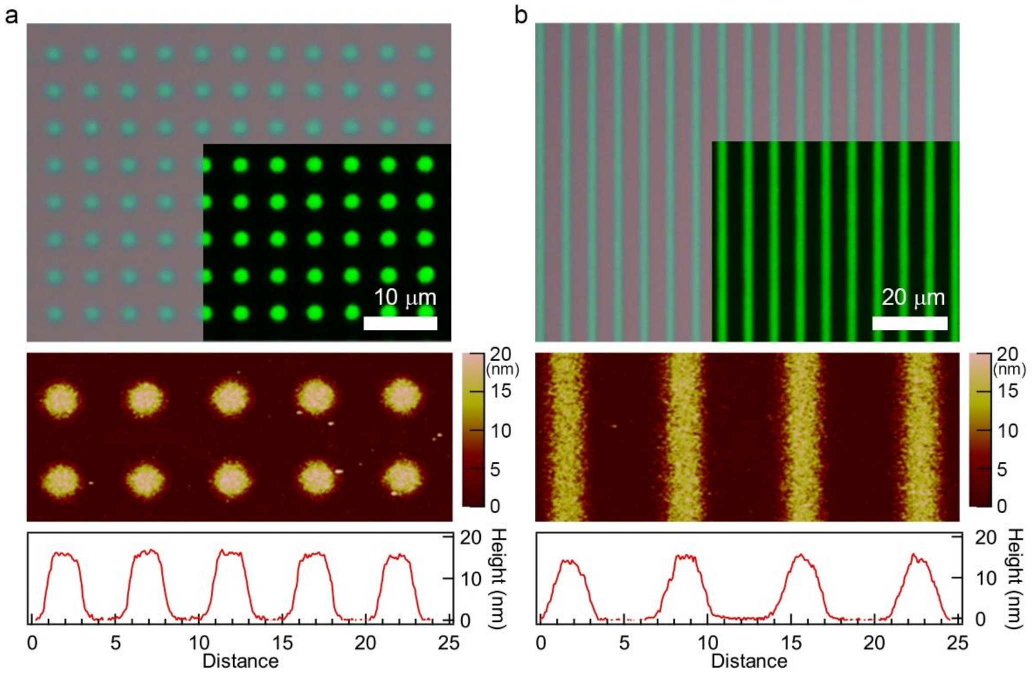

Supplementary Fig. 6 Optical and fluorescence (inset) images and AFM images with height profile a Dot and **b** line patterns based on 8-nm sized green-emitting CdSe/CdZnSeS QDs. These QD patterns were formed using 5 wt% of LiXer.

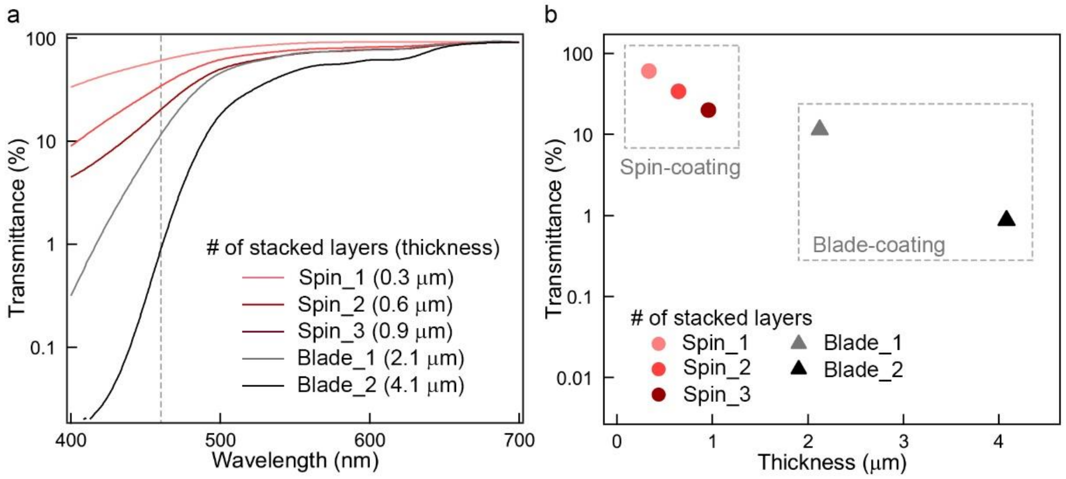

Supplementary Fig. 7 Transmission spectra of crosslinked QD films with different thicknesses a The series of crosslinked QD films were prepared by repeatedly applying spin-coating/crosslinking (red-series) or blade-coating/crosslinking (black-series) protocol. The grey dashed line visually guides a wavelength of 460 nm. **b** Values of %T at 460 nm from (a) plotted as a function of crosslinked film thickness.

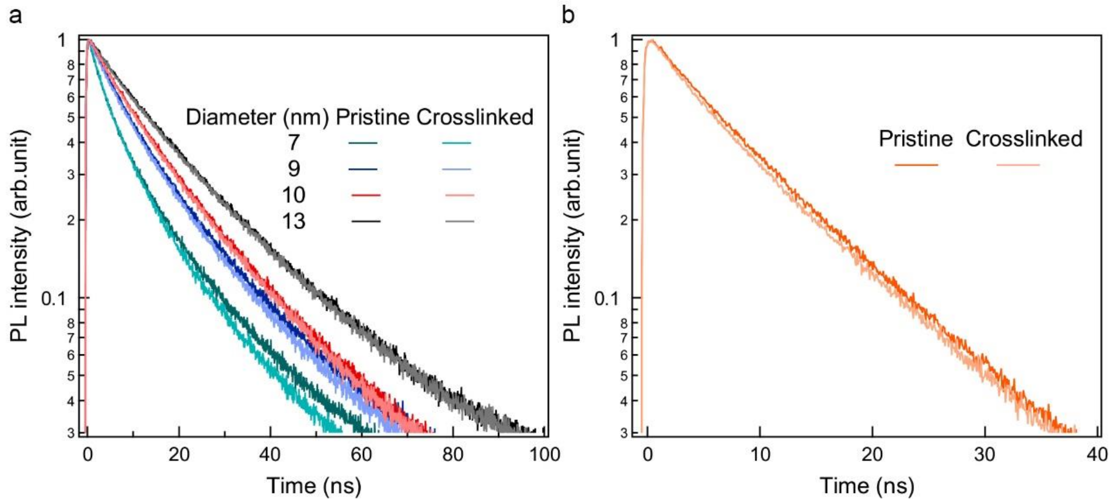

Supplementary Fig. 8 PL decay curves of pristine and crosslinked films of red-emitting QDs a 7-13nm sized red-emitting CdSe/CdZnS QDs and **b** 20-nm sized CdSe/CdZnSe/ZnSeS QDs. The crosslinked QD films were prepared with the optimum amount of LiXer.

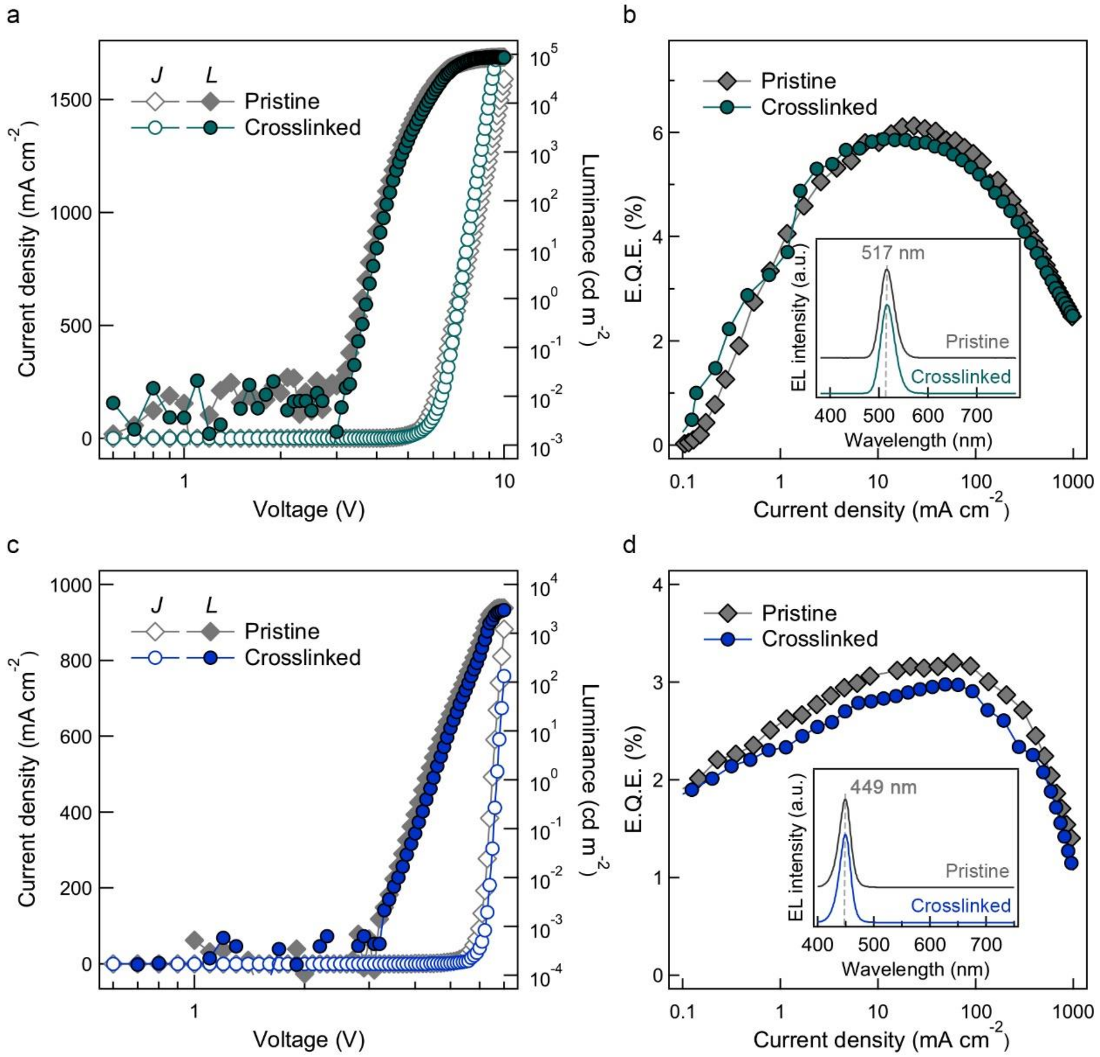

Supplementary Fig. 9 Electroluminescence characteristics of crosslinked green- and blue-emitting QD-LEDs a,c Current density (*J*)–voltage (*V*)–luminance (*L*) and **b,d** External quantum efficiency (EQE)-*J* characteristics of **a,b** green and **c,d** blue QD-LEDs employing pristine and crosslinked QD films. 2 wt% of LiXer was used for crosslinking. The inset in (b) and (d) shows EL spectra of each device. The EL spectrum of a pristine QD-LED is vertically shifted for visual clarity.

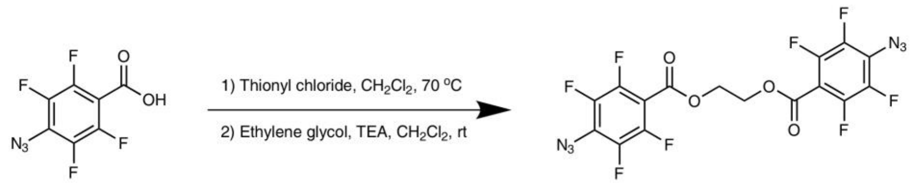

Supplementary Fig. 10 Schematic illustration of synthetic route to ethane-1,2-diyl bis(4-azido-2,3,5,6-tetrafluorobenzoate).

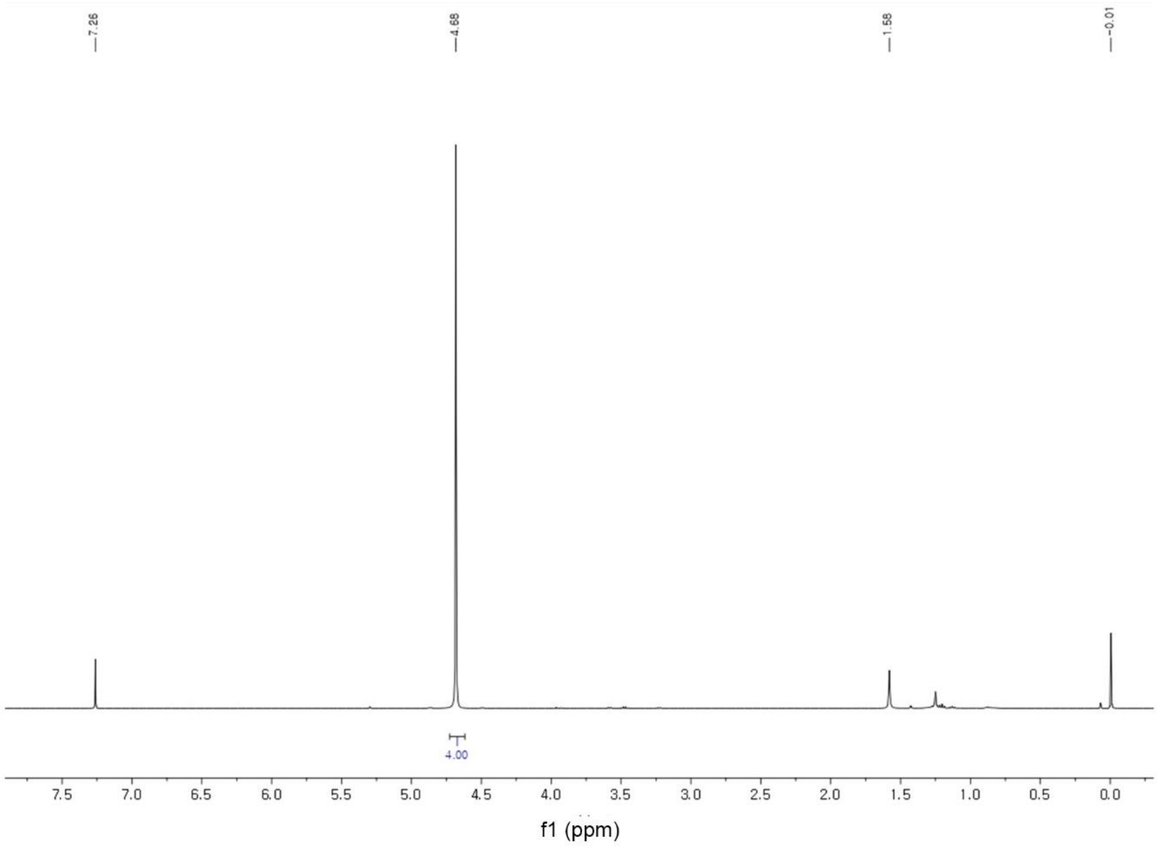

Supplementary Fig. 11 ^{1}H-NMR spectrum of ethane-1,2-diyl bis(4-azido-2,3,5,6-tetrafluorobenzoate).

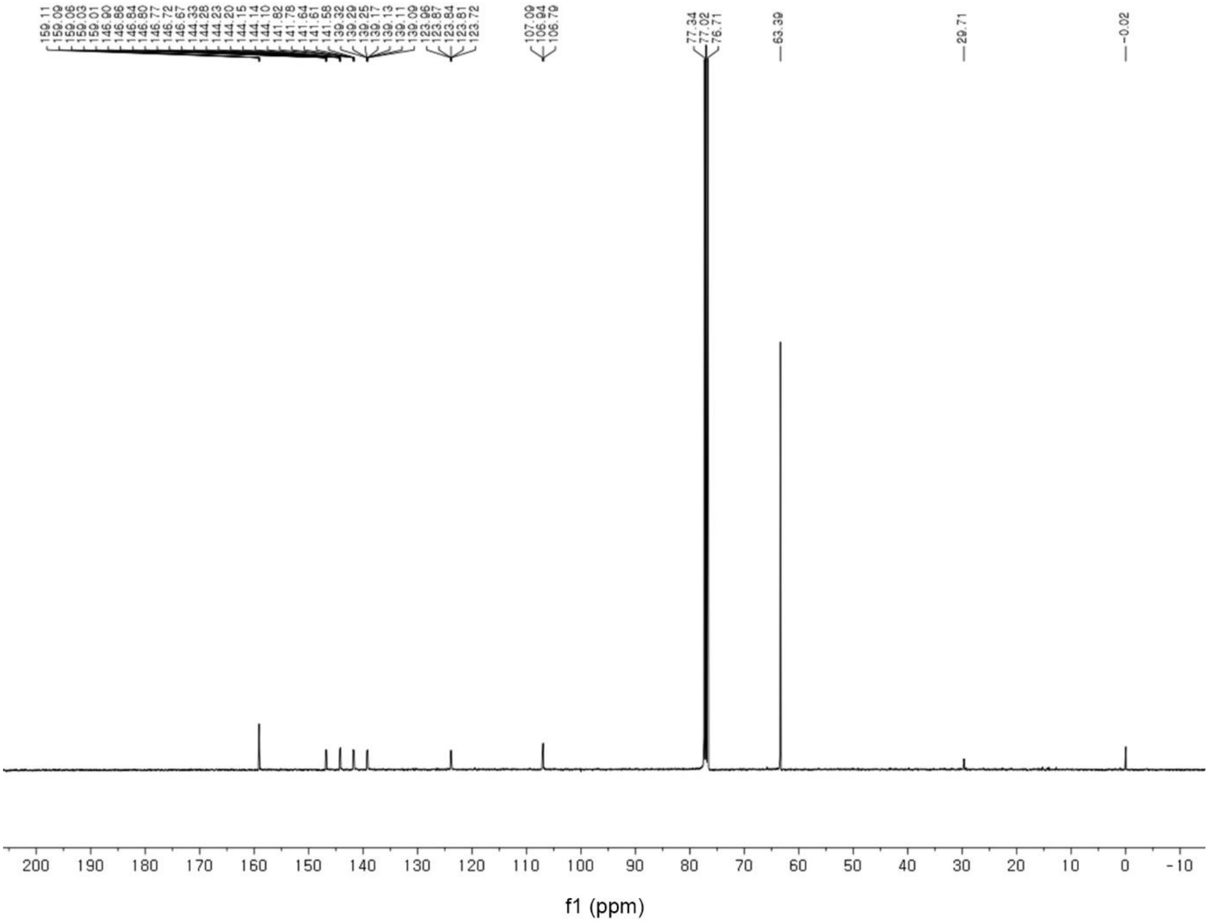

Supplementary Fig. 12 ^{13}C-NMR spectrum of ethane-1,2-diyl bis(4-azido-2,3,5,6-tetrafluorobenzoate).

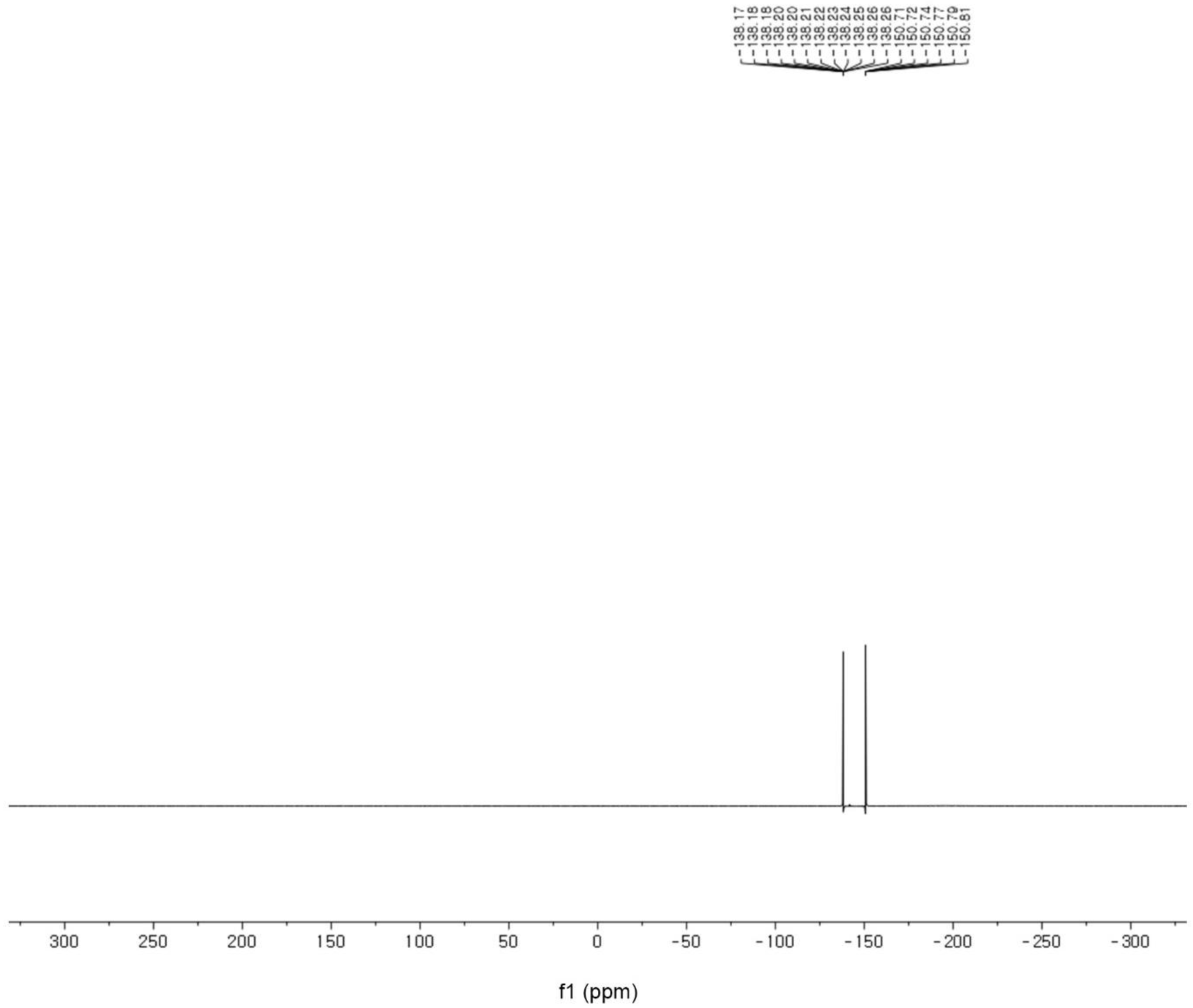

Supplementary Fig. 13 ^{19}F-NMR spectrum of ethane-1,2-diyl bis(4-azido-2,3,5,6-tetrafluorobenzoate).

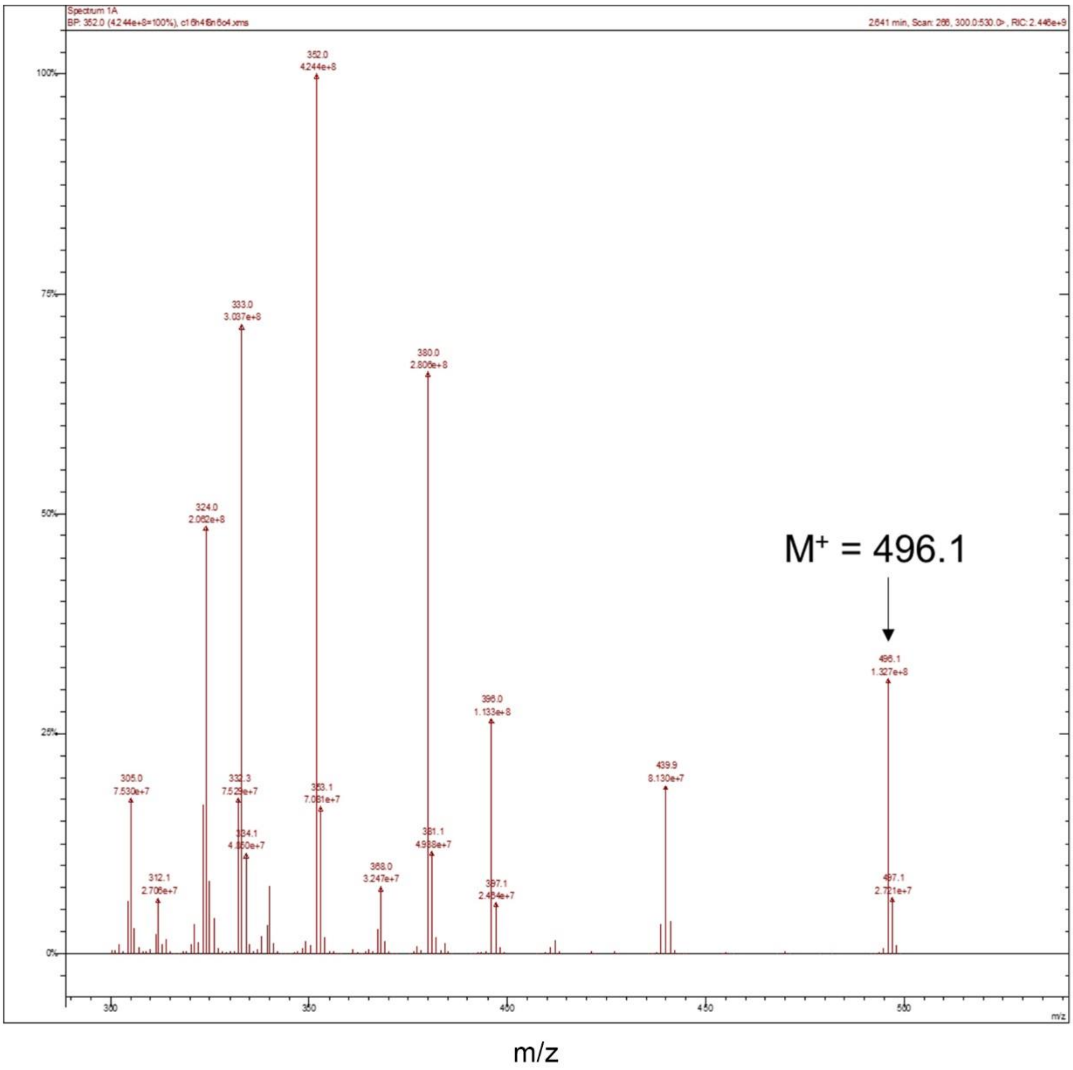

Supplementary Fig. 14 GC/MS spectrum of ethane-1,2-diyl bis(4-azido-2,3,5,6-tetrafluorobenzoate).

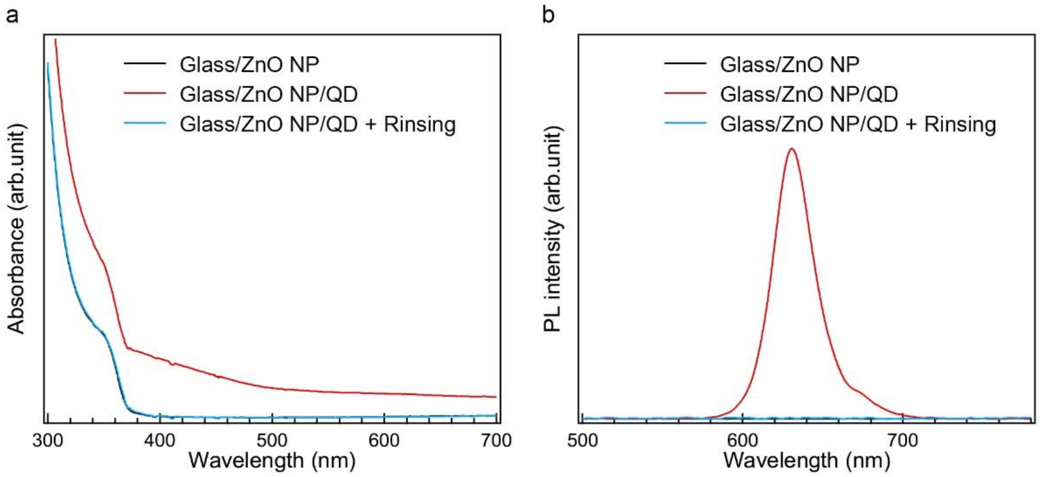

Supplementary Fig. 15 Effective removal of an uncrosslinked QD layer formed on the ZnO NP layer a Absorption and **b** photoluminescence (PL) spectra of a layer of ZnO NPs (black), a layer of QDs coated on the layer of ZnO NPs before (red) and after (blue) rinsing process. All samples were prepared on glass substrates. The samples were partially exposed to the UV source through a photomask and were rinsed with toluene. The blue curves were obtained from the section of the film that was covered with the photomask during the UV exposure, which is expected not to undergo the ligand crosslinking reaction.

Supplementary Discussion

Quantitative description of the photon-to-crosslink conversion process.

The entire incident photon-to-ligand crosslinking efficiency was estimated from the product of i) the number density of photons irradiated to the QD film, ii) the fraction of photons absorbed by LiXer, and iii) the efficiency of LiXer undergoing through the intended alkyl insertion. Each of these factors are obtained as follows.

i) The QD films with LiXer were irradiated to a 254 nm light source (0.4 mW cm^{-2}) for 5 sec. This corresponds to a dose of 2 mJ cm^{-2} (2.6×10^{15} photons cm^{-2}).

ii) We then estimated the fraction of photons absorbed by LiXer. For this estimation, the extinction coefficients for both QDs ($\varepsilon_{QD,254\ nm}$) and LiXer ($\varepsilon_{Lixer,254\ nm}$) at a wavelength of 254 nm were necessary. $\varepsilon_{QD,254\ nm}$ for the 13 nm-sized CdSe/CdZnS QDs we used in this study was measured from the absorptivity of CdSe/CdZnS QD dispersion with a given concentration in 1 cm length cuvette. The molar concentration of 13 nm-sized CdSe/CdZnS QD dispersion was obtained from the molar concentration of CdSe core (according to the method in previous report[R1]), which was used for the core/shell synthesis. The molar weight of QDs (MW_{QD}) was determined by measuring the weight of QDs at a given concentration of QD dispersion. This led us to acquire the molar extinction coefficient of 13 nm-sized CdSe/CdZnS QDs at 254 nm to be 5.3×10^7 $M^{-1}\cdot cm^{-1}$ and MW_{QD} of the 13 nm-sized CdSe/CdZnS QD (MW_{QD}) of 7.4×10^5 g mol^{-1}. On the other hand, the molar extinction coefficient ($\varepsilon_{Lixer,254\ nm}$) and the molecular weight (MW_{LiXer}) of LiXer were measured to be 3.6×10^4 $M^{-1}\cdot cm^{-1}$ and 496.23 g mol^{-1}, respectively.

Using the molar weights and the composition of LiXer in the film, we could estimate that there are 31 times higher number of LiXer molecules than that of QDs. Assuming that there are 6 nearest neighbor QDs for a single QD, ~10 (=(31/6)×2) LiXer molecules are involved in a single QD-QD linkage. Furthermore, from the molar extinction coefficients, molar weights, and composition of the film, we estimated that 2.06% of incident photons (254 nm) would be absorbed by LiXer. This indicates that 5.3×10^{13} photons are absorbed by LiXer per a given film area, *i.e*, 5.3×10^{13} photons absorbed by LiXer cm^{-2} (= 2.6×10^{15} photons $cm^{-2} \times 2.06\%$).

Suppose that the 13 nm-QDs in the film are randomly close-packed in two-dimensions, their number packing density is ~4.5×10^{11} QDs cm^{-2}. Thus, we could estimate the number of photons absorbed by numbers of LiXers attached on a single QD is equal to, *i.e*, 116 (=[5.3×10^{13} photons cm^{-2}]/[4.5×10^{11} QDs cm^{-2}]). Furthermore, based on the estimation above that there are 31 times more LiXers than QDs in the film, we could estimate that 3.7 photons are absorbed by a single LiXer molecule within the film.

iii) The efficiency of LiXer undergoing through the intended alkyl insertion reaction could be estimated by considering the quantum efficiency for photo-activation of the fluorinated phenyl azides in LiXer and the subsequent C-H insertion reaction based on the activated nitrene. It is known that halogenated phenyl azides have a very low probability of triplet or ring expansion reactions.[R2] Thus, we assumed that only the nitrenes in single state are generated. Based on the change in the areal intensity of azide peak from FT-IR measurement on the film after UV exposure, we estimated that this efficiency to be ~73% (Supplementary **Fig. 4**). The resulting singlet phenyl-nitrenes can undergo various reactions processes such as (i) ring expansion, (ii) intersystem crossing, and (iii) insertion/addition reaction. Since the surface ligands of QDs we examined are 1-dodecanethiol (DDT), which consists of simple hydrocarbon chain and a thiolate binding group, the possibility of other insertion reaction (*e.g.*, N-H insertion reaction or C=C cycloaddition reaction) is very low. Therefore, we assumed that nearly entire photo-activated nitrenes form chemical bonds with surface ligands of QDs through C-H insertion reaction.

Thus, based on the estimation above that there are ca. 10 molecules of LiXer reside in a single QD-QD linkage, we estimate that 7 molecules participate in the formation of a single QD-QD linkage.

Overall, under the given irradiation condition (2.6×10^{15} photons at 254 nm cm^{-2}) for 13 nm-CdSe/CdZnS QD films containing 2wt% of LiXer, 1.0×10^{13} number of crosslinks per area (1.0×10^{13} crosslinks cm^{-2}) are formed, which implies the formation of 7 chemical bonds between two adjacent QDs by LiXer.

Fabrication of μm-thick QD layers by repeating photo-crosslinking steps

Crosslinked QD films with thicknesses in hundreds of nm can be readily prepared by simply using a QD/LiXer solution of a higher concentration. For example, crosslinking a QD/LiXer film prepared by spin-coating (at 1000 rpm 30 sec) using a 100 mg mL^{-1} mixture solution yielded 340 nm-thick QD/LiXer films. Blade coating (adjusting the speed of a moving blade (5 cm sec^{-1}) and a blade gap (100 μm) with thermal annealing at 70 °C) of the same solution yielded 2 μm-thick QD/LiXer films.

More importantly, the thickness of films can be increased by executing the deposition process multiple times, due to the structural tolerance of the crosslinked QD films against the solvent used to deposit the QD films (e.g, toluene) – the underlying crosslinked QD films would not be dissolved during the processing of the upper QD films. For example, by applying the same spin-coating/crosslinking protocol repeatedly, the thickness of the resulting QD film increased from 340 nm (prepared from the primary deposition) to 645 nm after the secondary deposition step and to 950 nm after the tertiary deposition step. By applying the same bar-coating/crosslinking protocol repeatedly, the thickness of the resulting QD film increased from 2 μm (prepared from the primary deposition) to 4 μm after the secondary deposition step. Successive deposition of a crosslinked QD layer on top of a crosslinked layer resulted in thick QD films yielding a transmission at 460 nm wavelength reaching below 10% (Supplementary **Fig. 7**).

Supplementary Methods

Materials for synthesis. Zinc acetate ($Zn(ac)_2$, 99.99%), sulphur (S, 99.99%), selenium (Se, 99.99%), oleic acid (OA, 99%), and 1-octadecene (ODE, 99%) were purchased from Uniam. *n*-Trioctylphosphine (TOP, technical grade, 90%), zinc acetate dihydrate (> 98%), potassium hydroxide (99.99 %), methanol (99.9 %), 1-butanol (99.9 %) was purchased from Sigma Aldrich. Cadmium oxide (CdO, 99.9%) was purchased from Alfa Aesar. All chemicals are used as received.

Precursors for QD synthesis. For cadmium, zinc, selenium, and sulphur precursors, 0.5-M cadmium oleate ($Cd(OA)_2$) stock solution, 0.5-M zinc oleate ($Zn(OA)_2$) stock solution, 2-M TOPSe, and 2-M TOPS were prepared, respectively. For the preparation of $Cd(OA)_2$ stock solution, 100 mmol of CdO, 100 mL of OA, and 100 mL of ODE were placed in a flask and degassed at 110 °C. After filled with argon, the mixture was heated to 300 °C to form clear $Cd(OA)_2$ solution. For the preparation of $Zn(OA)_2$, 100 mmol of $Zn(ac)_2$ and an excess amount of OA (100 mL) were loaded in a flask and degassed at 160 °C for 1 h. After filling the flask with argon, 100 mL of degassed ODE was added to the flask. To prepare 2-M TOPS and 2-M TOPSe stock solutions, 100 mmol of S and Se was dissolved in 50 mL of TOP, respectively.

Synthesis of green-emitting CdSe/CdZnSeS QDs. CdSe/CdZnSeS QDs were prepared referring to the method used by Bae *et al.*[1] For a typical synthesis, 16 mmole of $Zn(OA)_2$ solution and 1.6 mmole of $Cd(OA)_2$, and 4 mL of ODE were placed into a flask and the mixture was degassed at 110 ℃. The reaction flask was filled back with argon, and the temperature was heated to 300 ℃. 0.6 mmol of TOPSe and 5 mmol of TOPS were rapidly injected to the flask and reacted for 2 min. For further growth, additional Cd, Zn, and S precursors were subsequently injected to the flask, and reacted for 10 min. Synthesized CdSe/CdZnSeS QDs were purified repeatedly *via* typical precipitation/redispersion method.

Synthesis of blue-emitting CdZnS/ZnS QDs. CdZnS/ZnS QDs were prepared via the reported procedure with minor modification.[2] As a typical synthesis, 1mmol of CdO, 10 mmol of $Zn(ac)_2$, and 7 mL of OA were loaded to a flask and the mixture was degassed at 160 ^oC. The flask was filled with argon gas before 15 mL of degassed ODE was added. After a clear solution of $Cd(OA)_2$ and $Zn(OA)_2$, the temperature of reaction flask was heated to 310 ^oC. 1.6 mmol of S dissolved in 2.4 mL of ODE was rapidly injected to the flask and reacted for 8 min. For further growth of ZnS shell, 8 mmol of S dissolved in 16 mL of ODE was slowly injected and reacted for 2 hours. Synthesized CdZnS/ZnS QDs were purified repeatedly *via* typical precipitation/redispersion method.

Synthesis of ZnO nanoparticles for device fabrication. ZnO nanoparticles were synthesised referring the method used by Lim *et al*.[3] First, 80 mL of 0.1-M zinc acetate dihydrate in methanol was placed in a three-neck round-bottom flask and heated to 60 °C. Then, 40 mL of 0.4-M potassium hydroxide with methanol was injected dropwise into the zinc acetate dihydrate solution with strong agitation. The mixture was kept at 60 °C for 2 h 15 min. After precipitation, the product was redispersed in 1-butanol.

Supplementary References

1. Bae, W. K. *et al.* Highly efficient green-light-emitting diodes based on CdSe@ZnS quantum dots with a chemical-composition gradient. *Adv. Mater.* **21**, 1690-1694 (2009).

2. Bae, W. K. *et al.* Deep blue light-emitting diodes based on $Cd_{1-x}Zn_xS$@ZnS quantum dots. *Nanotechnology* **20**, 075202 (2009).

3. Lim, J. *et al.* Highly efficient cadmium-free quantum dot light-emitting diodes enabled by the direct formation of excitons within InP@ZnSeS quantum dots. *ACS Nano* **7**, 9019-9026 (2013).
